# Supplementary material for: Listeria monocytogenes requires phosphotransferase systems to facilitate intracellular growth and virulence
Source: PLoS Pathog. 2025 Apr 15;21(4):e1012492. doi: 10.1371/journal.ppat.1012492 (PMC12052390; doi:10.1371/journal.ppat.1012492)
Supplement: S1 Table — Listed here are all bacterial strains used in this work. Those derived from prior works have been cited and those generated by us have been labeled under the ‘Reference’ column as ‘This Work’. Relevant strain numbers have been so that if others wish to use one of these strains in their own work it can be sourced from our strain repository. (DOCX) [file ppat.1012492.s001.docx]

| **Strain** | **Description** | **Reference** |
| --- | --- | --- |
| XL1-Blue | competent *E. coli* strain | (1) |
| SM10 | *E. coli* strain for conjugations into *L. monocytogenes;* Km^R^ | (1) |
| S17 | *E. coli* strain for conjugations into *L. monocytogenes;* Sp^R^ | (2) |
| 10403S [JDS1] | Background *L. monocytogenes* 10403s strain | (3) |
| JDS237 | PrfA* (G145S) | (4) |
| JDS2641 & MJF35 | Δ*uhpT* | This Work & (5) |
| JDS2642 & MJF112 | Δ*glpD*/Δ*golD* | This Work |
| JDS2643 & MJF121 | *△glpD/△golD/△uhpT* | This Work |
| JDS2644 & MJF279 | Δ*ptsI* | This Work |
| JDS2645 & MJF281 | *△glpD/△golD/△uhpT*/Δ*ptsI* | This Work |
| JDS2646 & MJF286 | Δ*ptsI::ptsI-C (pIMK2)* | This Work |
| JDS2647 & MJF288 | *△glpD/△golD/△uhpT*/Δ*ptsI::ptsI-C (pIMK2)* | This Work |
| JDS2648 & MJF223 | Δ*ptsH* | This Work & (6) |
| JDS2649 & MJF225 | *△glpD/△golD/△uhpT*/Δ*ptsH* | This Work |
| JDS2650 & MJF346 | PrfA* (G145S) Cleanly generated in JDS1 background | This Work |
| JDS2651 & MJF342 | *△glpD/△golD/△uhpT*::PrfA* (G145S) | This Work |
| JDS2652 & MJF348 | Δ*ptsI*::PrfA* (G145S) | This Work |

**Supplemental Table 1. Bacterial strains used in this study.**

**WORKS CITED**

1. Chen GY, McDougal CE, D’Antonio MA, Portman JL, Sauer JD. A Genetic Screen Reveals that Synthesis of 1,4-Dihydroxy-2-Naphthoate (DHNA), but Not Full-Length Menaquinone, Is Required for *Listeria monocytogenes* Cytosolic Survival. Swanson MS, editor. mBio [Internet]. 2017 May 3 [cited 2021 Dec 14];8(2). Available from: https://journals.asm.org/doi/10.1128/mBio.00119-17

2. Lauer P, Chow MYN, Loessner MJ, Portnoy DA, Calendar R. Construction, Characterization, and Use of Two Listeria monocytogenes Site-Speciﬁc Phage Integration Vectors. J BACTERIOL. 2002;184.

3. Bécavin C, Bouchier C, Lechat P, Archambaud C, Creno S, Gouin E, et al. Comparison of Widely Used Listeria monocytogenes Strains EGD, 10403S, and EGD-e Highlights Genomic Differences Underlying Variations in Pathogenicity. Casadevall A, editor. mBio. 2014 May;5(2):e00969-14.

4. Miner MD, Port GC, Freitag NE. Functional impact of mutational activation on the Listeria monocytogenes central virulence regulator PrfA. Microbiology. 2008 Nov 1;154(11):3579–89.

5. Chico-Calero I, Suárez M, González-Zorn B, Scortti M, Slaghuis J, Goebel W, et al. Hpt, a bacterial homolog of the microsomal glucose- 6-phosphate translocase, mediates rapid intracellular proliferation in Listeria. Proc Natl Acad Sci USA. 2002 Jan 8;99(1):431–6.

6. Mertins S, Joseph B, Goetz M, Ecke R, Seidel G, Sprehe M, et al. Interference of Components of the Phosphoenolpyruvate Phosphotransferase System with the Central Virulence Gene Regulator PrfA of *Listeria monocytogenes*. J Bacteriol. 2007 Jan 15;189(2):473–90.
