# Supplementary material for: Listeria monocytogenes requires phosphotransferase systems to facilitate intracellular growth and virulence
Source: PLoS Pathog. 2025 Apr 15;21(4):e1012492. doi: 10.1371/journal.ppat.1012492 (PMC12052390; doi:10.1371/journal.ppat.1012492)
Supplement: S2 Table — Below are primers used to generate the complement and clean-deletion constructs in pIMK2 or pLIM and pKSV7, respectively. Restriction enzyme cut sites used for homologous overlaps for Gibson Assembly have been provided. (PDF) [file ppat.1012492.s002.pdf]

Supplemental Table 2. Primers used in this study.

Primers Sequence	Description	Restriction Sites
TACGAATTCGAGCTCGGTACCCGGGG ATCCTTTGAATATAGCGATGGAAAAAAT GTTCTA	pKSV7 Construct *uhpT* deletion; Fragment 1 Forward	BamHI
TCT TCA CTC ATT GTT TTC AAC TTA TAG ATG TAA TGA CAT TAT AAT TTT CCT TTC CAG TGT	Construct *uhpT* deletion; Fragment 1 Reverse	N/A
ACACTGGAAAGGAAAATTATAATGTCAT TACATCTATAAGTTGAAAACAATGAGTG AAGA	Construct *uhpT* deletion; Fragment 2 Forward	N/A
GAC GGC CAG TGC CAA GCT TGC ATG CCT GCA GGT GCG GGC GCT GCG CTT GGT CCA GCT TTC G	pKSV7 Construct *uhpT* deletion; Fragment 2 Reverse	PstI
GGTACCCGGGGATCCCTACCAGCAAT GAAAGC	pKSV7 Construct *glpD* deletion; Fragment 1 Forward	BamHI
TTGAATTTTAATTATCTATCGTGTTGTAC CATTTTGTTTGC	Construct *glpD* deletion; Fragment 1 Reverse	N/A
GCAAACAAAATGGTACAACACGATAGA TAATTAAAATTCAA	Construct *glpD* deletion; Fragment 2 Forward	N/A
CTTGCATGCCTGCAGGCTTCGGTAGTA CTAACACC	pKSV7 Construct *glpD* deletion; Fragment 2 Reverse	PstI
GAGCTCGGTACCCGGGGATCCTAGAAT CAATCATTGATCTAAGC	pKSV7 Construct *golD* deletion; Fragment 1 Forward	BamHI
GAACTTTAAAGCTGAATACTTTATTTAAT AAAAGTCATTTTTCATTACCTCC	Construct *golD* deletion; Fragment 1 Reverse	N/A
GGAGGTAATGAAAAATGACTTTTATTAA ATAAAGTATTCAGCTTTAAAGTTC	Construct *golD* deletion; Fragment 2 Forward	N/A
CAGTGCCAAGCTTGCATGCCTGCAGG GCGTTTTACTGCCAG	pKSV7 Construct *golD* deletion; Fragment 2 Reverse	PstI

ATTCGAGCTCGGTACCCGGGGATCCTA GAGCATTGTCACTCGG	pLIM Construct *ptsI* deletion; Fragment 1 Forward	BamHI
GTCTTTATTCAGATATTATTCTGCAGTCT CTTTAGCCATTATTCAGCC	Construct *ptsI* deletion; Fragment 1 Reverse	N/A
GGCTGAATAATGGCTAAAGAGACTGCA GAATAATATCTGAATAAAGAC	Construct *ptsI* deletion; Fragment 2 Forward	N/A
CAAGCTTGCATGCCTGCAGGGATCCT GCACCAATCCCTTGAC	pLIM Construct *ptsI* deletion; Fragment 2 Reverse	BamHI
CCCATGGAAAAGGATCCATGGCTAAAG AGTTGAAAGGTATC	Construct *ptsI* complement; pIMK2 Vector Foreward	BamHI
ATATCGAATTCCTGCAGGTCTAGGTCTT TATTCAGATATTATTCTGC	Construct *ptsI* complement; pIMK2 Vector Reverse	PstI
ATTCGAGCTCGGTACCCGGGGATCCT GTTGCCTCTATCTTGCTG	pLIM Construct *ptsH* deletion; Fragment 1 Forward	BamHI
CCATTATTCAGCCAATCCTTCAAAACTT GCTTGTTCCATAATTTAC	Construct *ptsH* deletion; Fragment 1 Reverse	N/A
GTAAATTATGGAACAAGCAAGTTTTGAA GGATTGGCTGAATAATGG	Construct *ptsH* deletion; Fragment 2 Forward	N/A
GCAGGTCGACTCTAGAGGATCCAGATT CAAACATACCAATAAACATGTC	pLIM Construct *ptsH* deletion; Fragment 2 Reverse	BamHI
AGCTCGGTACCCGGGGATCCagactg cttatcaagcttccaaag	pLIM Construct PrfA* (G145S) Revertant FWD	BamHI
TTGCATGCCTGCAGGGATCCgtgatga tctagtagtggtttcacc	pLIM Construct PrfA* (G145S) Revertant RVS	BamHI
