# Supplementary material for: Listeria monocytogenes requires phosphotransferase systems to facilitate intracellular growth and virulence
Source: PLoS Pathog. 2025 Apr 15;21(4):e1012492. doi: 10.1371/journal.ppat.1012492 (PMC12052390; doi:10.1371/journal.ppat.1012492)
Supplement: S3 Table — The ‘Metabolite’ column shows scientific names for all metabolites screened using BioLog Phenotypic Microarrays. Fold usage of each metabolite for each strain [WT, ΔglpD/ΔgolD/ΔuhpT, and PrfA* (G145S)] is displayed relative the α-D-Glucose usage. Finally, p-values from comparisons of average metabolite usage per strain is shown for all metabolites. Comparison data was limited to WT v.s. ΔglpD/ΔgolD/ΔuhpT and WT v.s. PrfA*. (PDF) [file ppat.1012492.s003.pdf]

Supplemental Table 3. Results of BioLog PM1 and PM2A at 48 hours for WT, Δ*glpD*/Δ*golD*/Δ*uhpT*, and PrfA*.

	Fold Usage Normalized to α-D-Glucose			P-value (Student's T-Test)	
**Metabolite**	**WT**	**Δ*glpD*/Δ*golD*/Δ*uhpT***	**PrfA***	**WT v.s. Δ*glpD*/Δ*golD*/Δ*uhpT***	**WT v.s. PrfA***
Amygdalin	1.24	1.18	1.15	0.25	0.10
N-Acetyl-D-Glucosamine	1.06	0.99	1.05	0.30	0.76
Maltotriose	1.08	1.01	1.04	0.22	0.18
D-Trehalose	0.93	0.96	1.03	0.39	0.01
D-Glucose-6-Phosphate	0.17	0.17	1.02	0.97	<0.01
D-Fructose-6-Phosphate	0.21	0.19	1.01	0.68	<0.01
Beta-Methyl-D-Glucoside	1.03	1.02	1.01	0.78	0.72
alpha-Cyclodextrin	1.14	1.05	1.11	0.41	0.71
alpha-D-Glucose	1.00	1.00	1.00	N/A	N/A
D-Mannose	1.18	1.06	0.99	0.14	0.01
D-Cellobiose	0.98	0.96	0.99	0.70	0.83
Arbutin	1.17	1.15	1.08	0.53	0.06
D-Glucose-1-Phosphate	0.16	0.17	0.98	0.58	<0.01
Beta-Cyclodextrin	1.12	1.04	1.07	0.24	0.30
D-Fructose	1.06	0.91	0.96	0.25	0.38
Beta-D-Allose	1.12	1.04	1.04	0.25	0.16
Xylitol	1.18	1.06	1.03	0.30	0.24
Sailcin	1.03	1.03	1.05	0.98	0.63
Gamma-Cyclodextrin	0.98	0.99	1.02	0.88	0.55
N-Acetyl-Beta-D-Mannosamine	0.92	0.88	0.93	0.23	0.82
Glycerol	1.05	0.13	0.92	<0.01	0.31
L-Rhamnose	1.07	0.94	0.92	0.35	0.32
Gentiobiose	0.94	0.96	0.99	0.55	0.05
D-Psicose	1.05	0.92	0.89	0.31	0.20
Dihydroxy Acetone	1.15	0.99	0.94	0.24	0.14
D-Glucosamine	1.04	1.00	0.91	0.71	0.12
Uridine	0.93	0.80	0.81	0.45	0.48

Alpha-Methyl-D-Mannoside	1.17	1.08	0.86	0.36	0.12
L-Lyxose	0.89	0.70	0.75	0.21	0.36
2-Deoxy-D-Ribose	0.86	0.74	0.77	0.34	0.44
alpha-Methyl-D-Glucoside	1.23	0.55	0.79	<0.01	0.02
D-Arabitol	0.99	0.95	0.74	0.77	0.01
D-Ribose	0.78	0.68	0.65	0.41	0.27
Maltose	1.12	1.07	0.64	0.25	<0.01
alpha-D-Lactose	1.24	1.14	0.59	0.30	<0.01
D-Malic Acid	0.14	0.16	0.58	0.09	0.39
D-Xylose	0.65	0.54	0.54	0.47	0.49
Acetic Acid	0.59	0.57	0.51	0.69	0.35
5-Keto-D-Gluconic Acid	0.60	0.53	0.52	0.27	0.20
Capric Acid	0.46	0.43	0.46	0.85	0.98
Inosine	0.57	0.48	0.40	0.51	0.23
N-Acetyl-D-Glucosaminitol	0.53	0.43	0.43	0.27	0.25
Acetoacetic Acid	0.41	0.37	0.38	0.73	0.80
Sorbic Acid	0.48	0.44	0.42	0.39	0.23
Dextrin	0.70	0.57	0.42	0.33	0.07
L-Arabinose	0.42	0.37	0.36	0.51	0.47
m-Tartaric Acid	0.14	0.16	0.35	0.08	0.42
Adenosine	0.40	0.35	0.34	0.57	0.48
D-Arabinose	0.48	0.40	0.34	0.21	0.04
alpha-keto-Butyric Acid	0.48	0.48	0.30	1.00	<0.01
Palatinose	0.38	0.33	0.28	0.25	0.02
Mucic Acid	0.31	0.39	0.25	0.50	0.59
D-Fucose	0.28	0.30	0.27	0.69	0.78
Beta-Methyl-D-Galactoside	0.30	0.28	0.26	0.81	0.54
Putrescine	0.20	0.18	0.27	0.71	0.28
D-Tagatose	0.36	0.32	0.26	0.59	0.18
Thymidine	0.24	0.27	0.23	0.50	0.73
3-Methyl Glucose	0.32	0.31	0.25	0.94	0.50
Adonitol	0.25	0.23	0.22	0.70	0.66

Oxalic Acid	0.29	0.31	0.24	0.79	0.22
Glyoxylic Acid	0.24	0.26	0.22	0.88	0.65
Sedoheptulosan	0.25	0.24	0.24	0.88	0.84
Glucuronamide	0.27	0.24	0.21	0.15	0.03
D,L-Octopamine	0.22	0.20	0.23	0.65	0.90
2-Deoxy-Adenosine	0.26	0.29	0.20	0.56	0.29
Pyruvic Acid	0.26	0.27	0.20	0.69	0.01
Oxalomalic Acid	0.27	0.24	0.22	0.59	0.31
2,3-Butanedione	0.25	0.22	0.22	0.38	0.37
Mannan	0.24	0.21	0.22	0.57	0.67
L-Histidine	0.23	0.21	0.21	0.57	0.41
Turanose	0.31	0.25	0.21	0.30	0.09
Pectin	0.29	0.29	0.21	0.86	0.06
Sebacic Acid	0.60	0.22	0.20	0.21	0.19
L-Sorbose	0.23	0.22	0.20	0.88	0.56
alpah-Keto-Valeric Acid	0.24	0.21	0.19	0.60	0.35
Sec-Butylamine	0.22	0.22	0.19	0.94	0.37
Beta-Methyl-D-Xyloside	0.21	0.24	0.19	0.54	0.66
L-Fucose	0.22	0.21	0.17	0.80	0.27
N-Acetyl-Neuaminic Acid	0.21	0.20	0.19	0.48	0.31
Methyl Pyruvate	0.20	0.21	0.17	0.46	0.02
Propionic Acid	0.20	0.24	0.17	0.32	0.20
3-0-Beta-D-Galactopyranosyl-D-Arabinose	0.26	0.24	0.18	0.67	0.07
L-Alanyl-Glycine	0.25	0.26	0.16	0.34	<0.01
L-Galactonic Acide-gamma-lactone	0.19	0.19	0.16	0.80	0.23
Inulin	0.34	0.27	0.18	0.46	0.11
L-Pyroglutamic Acid	0.21	0.19	0.17	0.49	0.30
D-Melezitose	0.23	0.21	0.18	0.67	0.31
L-Lactic Acid	0.26	0.25	0.16	0.83	0.03
L-Alanine	0.19	0.21	0.16	0.59	0.26
Tween 40	0.18	0.19	0.15	0.51	0.14
D-Sorbitol	0.21	0.18	0.15	0.08	0.02

D-Galactose	0.20	0.17	0.15	0.57	0.29
D,L-alpha-glycerol-phosphate	0.17	0.17	0.15	0.85	0.38
D-Galactonic Acid-gamma-Lactone	0.16	0.16	0.15	0.55	0.57
alpha-Methyl-D-Galactoside	0.18	0.18	0.15	0.99	0.49
D-Glucuronic Acid	0.19	0.19	0.15	0.89	0.20
L-Alaninamide	0.20	0.19	0.16	0.94	0.23
Butyric Acid	0.20	0.26	0.16	0.47	0.19
2-Aminoethanol	0.17	0.18	0.14	0.44	0.14
L-Methionine	0.20	0.16	0.16	0.46	0.55
alpha-Hydroxy Butyric Acid	0.21	0.21	0.14	0.96	0.13
D-Galacturonic Acid	0.18	0.18	0.14	0.93	0.04
L-Arabitol	0.19	0.16	0.16	0.34	0.26
Maltitol	0.22	0.18	0.16	0.47	0.29
D-Mannitol	0.17	0.17	0.14	0.83	0.20
Tween 80	0.17	0.17	0.14	1.00	0.09
m-Hydroxy Phenyl Acetic Acid	0.17	0.18	0.14	0.57	0.06
Glycogen	0.21	0.19	0.15	0.49	0.09
Dulcitol	0.17	0.16	0.14	0.69	0.19
Glycyl-L-Proline	0.22	0.19	0.14	0.17	0.02
Beta-Methyl-D-Glucuronic Acid	0.20	0.18	0.15	0.64	0.24
Lactitol	0.20	0.19	0.15	0.81	0.26
Delta-Amino Valeric Acid	0.16	0.15	0.15	0.70	0.75
L-Glucose	0.20	0.18	0.15	0.58	0.23
L-Leucine	0.16	0.15	0.15	0.55	0.62
Glycine	0.18	0.17	0.15	0.68	0.27
Caproic Acid	0.18	0.17	0.15	0.58	0.20
L-Proline	0.18	0.18	0.14	0.94	0.07
Succinic Acid	0.17	0.18	0.14	0.72	0.06
D-Gluconic Acid	0.17	0.18	0.14	0.61	0.12
Negative Control	0.18	0.17	0.14	0.84	0.03
Negative Control	0.18	0.16	0.15	0.49	0.43
3-Hydroxy-2-Butanone	0.21	0.17	0.15	0.41	0.27

Bromo Succinic Acid	0.15	0.18	0.13	0.35	0.21
D-Alanine	0.16	0.17	0.13	0.55	0.21
2-Hydroxy Benzoic Acid	0.16	0.17	0.15	0.62	0.39
L-Aspartic Acid	0.17	0.16	0.13	0.50	0.03
Apha-Hydroxy Glutaric Acid-gamma-lactone	0.16	0.18	0.13	0.33	0.24
p-Hydroxy Phenyl Acetic Acid	0.16	0.17	0.13	0.58	0.01
i-Erythritol	0.19	0.17	0.15	0.26	0.05
Beta-Hydroxy Butyric Acid	0.20	0.19	0.15	0.91	0.16
Sucrose	0.16	0.17	0.13	0.65	0.22
D-Threonine	0.14	0.15	0.13	0.63	0.51
D-Ribono-1,4-Lactone	0.16	0.16	0.15	0.96	0.26
Tyramine	0.16	0.17	0.13	0.57	0.18
Glycolic Acid	0.15	0.17	0.13	0.16	0.10
Chondroitin Sulfate C	0.19	0.16	0.15	0.33	0.18
L-Threonine	0.16	0.18	0.13	0.44	0.08
D-Saccharic Acid	0.17	0.18	0.13	0.87	0.11
D-Melibiose	0.17	0.18	0.13	0.91	0.10
4-Hydroxy Benzoic Acid	0.21	0.19	0.14	0.46	0.06
D-Raffinose	0.19	0.16	0.14	0.34	0.11
Alpha-Keto-Glutaric Acid	0.17	0.16	0.13	0.86	0.17
Gelatin	0.21	0.18	0.14	0.42	0.11
L-Lysine	0.18	0.17	0.14	0.73	0.26
Stachyose	0.19	0.18	0.14	0.65	0.19
L-Serine	0.17	0.17	0.13	0.96	0.01
Mono Methyl Succinate	0.16	0.17	0.13	0.39	0.01
Laminarin	0.17	0.18	0.14	0.92	0.15
Glycyl-L-Aspartic Acid	0.18	0.17	0.13	0.56	0.14
D-Aspartic Acid	0.15	0.16	0.13	0.61	0.17
Lactulose	0.17	0.21	0.13	0.42	0.15
L-Glutamic Acid	0.17	0.18	0.13	0.74	0.06
Tricarballylic Acid	0.15	0.17	0.13	0.31	0.09
Citraconic Acid	0.18	0.18	0.14	0.95	0.27

1,2-Propanediol	0.16	0.17	0.13	0.81	0.25
L-Valine	0.17	0.17	0.14	1.00	0.27
Formic Acid	0.15	0.17	0.13	0.35	0.10
Phenylethylamine	0.15	0.16	0.13	0.39	0.30
N-Acetyl-L-Glutamic Acid	0.18	0.17	0.14	0.87	0.22
Fumaric Acid	0.15	0.17	0.13	0.30	0.08
Citric Acid	0.14	0.16	0.13	0.29	0.19
Succinamic Acid	0.18	0.16	0.14	0.55	0.17
N-Acetyl-D-Galactosamine	0.16	0.16	0.14	0.96	0.38
Glycyl-L-Glutamic Acid	0.17	0.17	0.13	0.75	0.03
L-Homoserine	0.17	0.16	0.14	0.71	0.16
Tween 20	0.15	0.14	0.12	0.91	0.44
L-Glutamine	0.15	0.16	0.12	0.18	0.07
L-Isoleucine	0.17	0.16	0.14	0.92	0.31
myo-Inositol	0.15	0.17	0.12	0.15	0.08
L-Ornithine	0.17	0.17	0.14	0.94	0.10
2,3-Butanediol	0.19	0.17	0.14	0.58	0.12
Glycolic Acid	0.17	0.17	0.14	0.93	0.18
D-Serine	0.15	0.14	0.12	0.90	0.13
Itaconic Acid	0.16	0.14	0.14	0.47	0.36
L-Asparagine	0.16	0.17	0.12	0.79	0.12
Hydroxy-L Prline	0.18	0.17	0.14	0.56	0.10
Quinic Acid	0.18	0.17	0.13	0.65	0.03
Melibionic Acid	0.23	0.20	0.13	0.82	0.32
gamma-Amino Butyric Acid	0.17	0.17	0.13	0.97	0.24
D,L-Malic Acid	0.16	0.17	0.12	0.58	0.06
D-Glucosaminic Acid	0.15	0.17	0.12	0.21	0.10
L-Malic Acid	0.15	0.15	0.12	0.39	<0.01
L-Arginine	0.17	0.16	0.13	0.48	0.11
D,L-Carnitine	0.17	0.17	0.13	0.88	0.10
Acetamide	0.18	0.17	0.13	0.71	0.10
D-Tartaric Acid	0.17	0.17	0.13	0.94	0.14

Citramalic Acid	0.17	0.17	0.13	0.95	0.17
L-Phenylalanine	0.17	0.16	0.13	0.85	0.11
D-Lactic Acid Methyl Ester	0.16	0.16	0.13	0.97	0.07
L-Tartaric Acid	0.17	0.17	0.13	0.88	0.02
Malonic Acide	0.17	0.16	0.12	0.52	0.10
